# Tanshinone IIA induces intrinsic apoptosis in osteosarcoma cells both *in vivo* and *in vitro* associated with mitochondrial dysfunction

**DOI:** 10.1038/srep40382

**Published:** 2017-01-20

**Authors:** Sheng-Teng Huang, Chao-Chun Huang, Wen-Liang Huang, Tsu-Kung Lin, Pei-Lin Liao, Pei-Wen Wang, Chia-Wei Liou, Jiin-Haur Chuang

**Affiliations:** 1 Department of Chinese Medicine, China Medical University Hospital, Taichung, Taiwan; 2 School of Chinese Medicine, China Medical University, Taichung, Taiwan; 3 Division of General Surgery, Ministry of Health and Welfare Pingtung Hospital, Pingtung, Taiwan; 4 Department of Neurology and Mitochondrial Research Unit, Kaohsiung Chang Gung Memorial Hospital and Chang Gung University College of Medicine, Kaohsiung, Taiwan; 5 Division of Pediatric Surgery and Mitochondrial Research Unit, Kaohsiung Chang Gung Memorial Hospital and Chang Gung University College of Medicine, Kaohsiung, Taiwan; 6 Department of Internal Medicine and Mitochondrial Research Unit, Kaohsiung Chang Gung Memorial Hospital and Chang Gung University College of Medicine, Kaohsiung, Taiwan

## Abstract

Tanshinone IIA (Tan IIA), a phytochemical derived from the roots of Salvia miltiorrhiza, has been shown to inhibit growth and induce apoptosis in various cancer cells. The association of its inhibitory effect on the primary malignant bone tumor, osteosarcoma, with mitochondrial dysfunction remains unclear. This study aimed to investigate the anti-proliferative effects of Tan IIA on human osteosarcoma 143B cells both *in vitro* and *in vivo*. Administration of Tan IIA to NOD-SCID mice implanted with 143B cells led to significant inhibition of tumor development. The inhibition of proliferation, migration, and invasion was observed in 143B cells treated with Tan IIA. The tumor proliferation markers, Ki67 and PCNA, were suppressed and apoptosis by TUNEL assay was activated respectively. Apoptosis in the Tan IIA-treated 143B cells and xerograft mice was associated with the activation of caspase cascade via the modulation of Bcl-2 family. The CD31 was inhibited in Tan IIA-treated xenografts to indicate anti-neovasculization. Tan IIA administration resulted in a significant decrease in the mitochondrial fusion proteins, Mfn1/2 and Opa1, as well as an increase in the fission protein Drp1. We concluded that mitochondrial dysfunction associated with dynamic change was involved in apoptosis and anti-angiogenesis elicited by Tan IIA.

The osteosarcoma is considered to be an aggressive malignant neoplasm with poor prognosis. Most of the patients with osteosarcoma are treated by chemotherapy in combination with aggressive surgical resection. However, the recurrent rate of this cancer is still around 30–40% and the 10-year survival rate is decreased to 20–30% with lung metastasis^1^. High dose of chemotherapeutic agents is restricted due to their systemic unbearable side effects. Therefore, the development of new regimens to inhibit recurrent and refractory osteosarcoma as well as the investigation of relevant mechanisms to understand antitumor effect is critical and urgent.

Apoptosis plays a critical role in chemotherapies against a variety of cancers. Apoptosis is known to be regulated by pro-apoptotic and anti-apoptotic B-cell lymphoma 2 (Bcl-2) family proteins and mediated by the activation of caspase cascade through two major pathways, namely, the mitochondria-dependent intrinsic pathway and the death receptor-mediated extrinsic pathway^2^. Angiogenesis is a crucial process in supplying the required nutrients and oxygen during the development, growth as well as progression and metastasis of cancer. It allows metastatic tumor cells to leak into the circulation and to reside in the organs by breaking through the basement membrane and endothelial cells (EC) to come into a new vessel^3^,^4^. Vascular branching signals released from ECs regulate mitochondrial biogenesis in the neovascularization. Vascular endothelial growth factor (VEGF) plays an essential role to stimulate mitochondrial biogenesis via protein kinase B (Akt) -dependent signaling, whereas the silencing of sirtuin 1 (SIRT1), a negative regulator of the Notch pathway, inhibits mitochondrial biogenesis in ECs^5^. On the other hand, the exposure of ECs to high levels of stimuli such as reactive oxygen species (ROS) production and calcium (Ca^2+^) overload will augment mitochondrial fission and/or reduces mitochondrial fusion and result in mitochondrial fragmentation. Ultimately, EC dysfunction and death are induced^5^. Thus, finding reagents to induce apoptosis and/or inhibit angiogenesis targeting on mitochondrion has become a main focus in cancer treatment^6^.

The shape of mitochondria can be regulated in various physiological conditions and pathological disorders via a balance of dynamic mitochondrial fusion and fission processes^7^. Fusion is mediated by the production of tubular or net-like structures promoted by homotypic/heterotypic interactions of the mitofusin1 (Mfn 1) and Mfn 2 dynamin-related guanosine-5′-triphosphatase (GTPases) at the outer mitochondrial membrane (OMM) of adjacent mitochondria and optic atrophy type 1 (Opa-1), also a dynamin-related GTPase, at the inner mitochondrial membrane (IMM). Fission of mitochondria requires the recruitment of a different dynamin-related GTPase, dynamin-related protein 1 (Drp1), and a tetratricopeptide domain protein, fission 1 protein (Fis1), to the OMM to induce the generation of smaller organelles^8^,^9^. Recently, mitochondrial fusion/fission machinery has been shown to participate in outer mitochondrial membrane permeability and induce apoptosis through intrinsic pathway to release cytochrome c from the inter-membrane space to initiate caspase activation in the cytosol^10^. Mitochondrial dynamics are also pertinent to endothelial cell function to affect angiogenesis in response to VEGF and Akt-dependent activation by inhibiting fusion process^11^. These findings indicate that mitochondrial dynamics related to fusion and fission is directly involved in not only apoptosis but also angiogenesis.

Danshen, the dried root of *Salvia miltiorrhiza*Bunge, is a well-known herb in traditional Chinese medicine that is commonly used as preventative or therapeutic preparations for coronary heart diseases, vascular diseases, stroke, hyperlipidemia, arthritis, hepatitis and cancer in clinical application^12^. Tanshinone IIA (Tan IIA) is one of the major diterpenes extracted from Danshen with anti-oxidative^13^, anti-inflammatory^14^ and anti-atherosclerotic^15^ effects. Our previous study^3^ found that Tan IIA with anti-angiogenic effects, both *in vivo* and *in vitro*, was associated with modulation with the secretion between matrix metalloproteinase 2 (MMP-2) and TIMP metallopeptidase inhibitor 2 (TIMP-2) in an opposite way and resulted in the decreased MMP-2 activity of vascular endothelial cells without cytotoxicity. In addition, Tan IIA was reported to inhibit growth through cell cycle arrest and apoptosis via intrinsic and extrinsic pathways on various cancer cell lines^16^,^17^,^18^. These studies demonstrated that Tan IIA could inhibit cell growth by down-regulating cyclins and cyclin-dependent kinases (CDKs) and even trigger apoptosis through the activation of selective caspase family members. Zhou *et al*.^19^ found that Tan IIA could disrupt the mitotic spindle to arrest cancer cell growth in mitotic phase and subsequently induced apoptosis through intrinsic pathway. Moreover, Tan IIA also induced apoptosis in xenograft tumors, including colon cancer in male severe combined immune-deficient (SCID) mice^20^ and breast cancer in female nude mice^21^. All of anti-cancerous activities of Tan IIA are proposed to be associated with such signal transduction pathways as PI3K/Akt, p38 mitogen-activated protein kinases (MAPK) and activator protein 1 (AP-1)^12^.

Tan IIA plays an important role to inhibit cancer cell growth by inducing apoptosis and angiogenesis. However, apoptosis and angiogenesis are highly associated with mitochondrial dysfunction. The relationship between these biological function and mitochondria affected by Tan IIA remains still not unclear. Based on its *in vitro* effects on inducing apoptosis and inhibiting proliferation, migration, and invasion, Tan IIA is proposed to be a potential anti-osteosarcoma (MG-63 cells) drug^22^. However, the association between its anti-osteosarcoma effect and mitochondrial dysfunction has not been demonstrated *in vivo*. The aim of this study is to elucidate the mitochondrial dynamics and associated morphological changes in osteosarcoma 143B cells treated by Tan IIA using *in vitro* and *in vivo* studies.

## Results

### Effect of Tan IIA on cell viability, cell migration and invasion of osteosarcoma 143B cells

**Figure 1:**
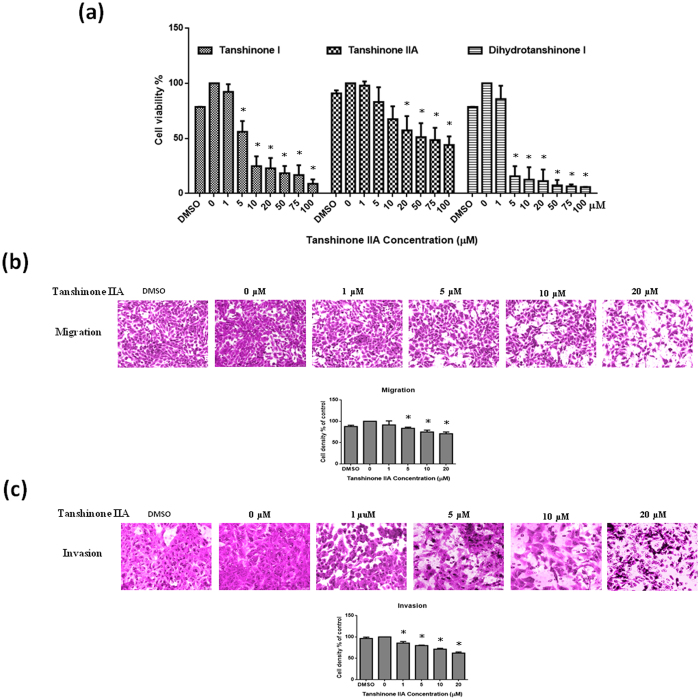
Tan IIA dose-dependently inhibited the cell viability, migration and invasion of 143B cells. (**a**) Cells were treated with concentrations from 0-100 µM of Tan IIA, tanshinone I and dihydrotanshinone I for 24 hours. The cell viability was determined by standard MTT assay. Cells pretreated 24 hours with concentrations of Tan IIA (0-20 µM) were allowed to migrate or invade for 2 or 24 hours by transwell or matrix-coated transwell filter assays. Representative photos indicated migration (**b**) and invasion (**c**) of 143B cells induced with or without Tan IIA treatment. The cells on the lower surface of the filter were examined under contract microscope (HPF) (100X). The inhibitory effect of both migration and invasion by Tan IIA on 143B cells was demonstrated dose-dependently. The bar value is the mean ± SD of three independent experiments in duplicate. Asterisks mean statistical significance in comparison with the vehicle control (P < 0.05).

Tan IIA, tanshinone I and dihydrotanshinone I significantly reduced the 143B cell viability in a dose-dependent manner (Fig. 1a). We also examined whether Tan IIA could exert any effect on the migration and invasion of 143B cells as analyzed by transwell migration assay and matrix invasion assay. The inhibitory effect of Fig. 1b and c showed that Tan IIA dose-dependently inhibited cell migration and invasion. It clearly indicated that Tan IIA could significantly inhibit the process of cell proliferation and migration and matrix invasion of 143 B cells *in vitro*.

### Effect of Tan IIA on tumor growth and major organs in NOD-SCID mice

**Figure 2:**
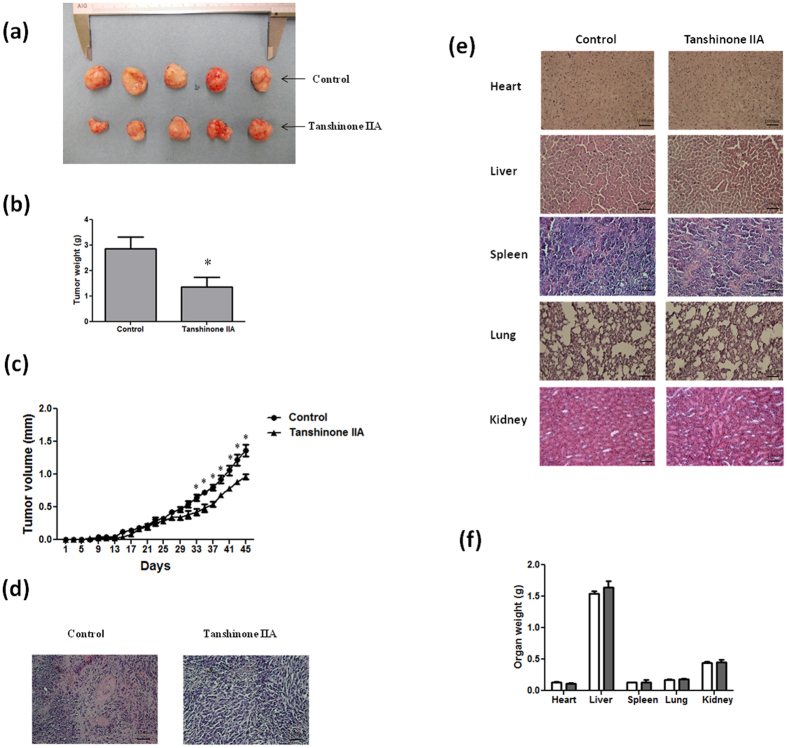
Effect of Tan IIA on the tumor growth and major organs in NOD-SCID mice with or without143B transplants. The tumor mass was dissected from each mouse 45 days after the implantation and weighed. Tan IIA (20 mg/kg) inhibited tumor growth measured in solid tumors (**a**), tumor weight (**b**) as well as tumor volume (**c**). Histopathologic analysis of the tumors (**d**) and major organs including heart, liver, spleen, lungs and kidneys (e) in Tan IIA group compared to those in the control group by Hematoxylin and Eosin stain, original magnification ×200. (**f**) The organ weight had no significant differences between Tan IIA-treated and non-treated groups. Arrows indicates osteosarcoma. Values are mean ± SD (n=15). An asterisk demonstrates the significant difference (p < 0.05) between control and Tan IIA-treated groups.

To investigate the *in vivo* effects of Tan IIA on tumor growth in mice, NOD-SCID mice were treated with or without subcutaneous injection of Tan IIA (20 mg/kg). Tumor development was carefully examined one week after the injection of 143B cells into the posterior side of NOD-SCID mice. During the period of 45 days of injection of Tan IIA, we found that Tan IIA significantly inhibited tumor size and tumor weight compared to the control group (Fig. 2a and b). The tumor volume is increased in a time-dependent manner. However, the tumor growth was significantly slower in Tan IIA-treated mice compared to control group (Fig. 2c). To verify the changes of tumor morphology between control and Tan IIA groups with H & E staining, a significant proliferation of osteoid with a high density of malignant cells in the vehicle control mice but not in Tan IIA treatment mice (Fig. 2d) was observed. Altogether it indicated that the administration of Tan IIA delayed the onset of tumor development in mice as well as suppressed the increase of tumor growth. To determine the potential toxic effects of Tan IIA on mice, the major organs, including liver, heart, lungs, spleen and kidneys, were removed and weighted. As shown in Fig. 2e, H and E staining revealed no significant differences between control and Tan IIA group. Also, there were no significant differences in body weight and internal organs of mice between these two groups (Fig. 2f). It is worth to note that, among all the sections observed, no evidence of tumor metastasis in the mice injected with osteosarcoma 143B cells was found, which was different from our in vitro observation with migration and invasion. The possible reason to explain this phenomenon could be the injection site of 143B cells onto subcutaneous tissue instead of bone marrow.

### Tan IIA exerted anti-proliferative, anti-angiogenic and pro-apoptotic effects

**Figure 3:**
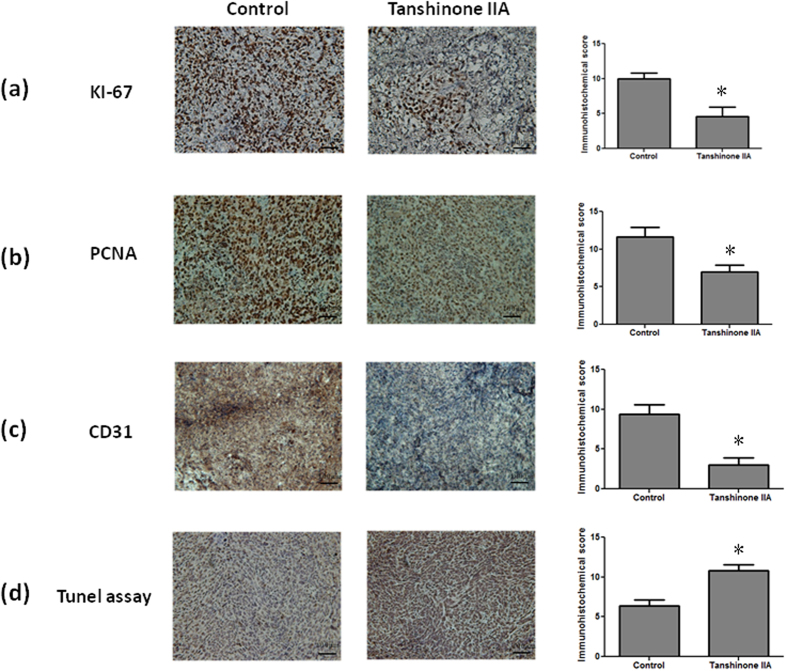
Effect of Tan IIA treatment on markers of proliferation, angiogenesis and apoptosis in tumors of NOD-SCID mice implanted with 143B cells. The proliferating cells in the tumor specimens from the different groups were detected by Ki67 (**a**) and PCNA (**b**) immunohistochemistry. CD31 (**c**), markers of angiogenesis, in the tumor specimens were also detected in these two groups. All of the markers of proliferation and angiogenesis were significantly inhibited in Tan IIA-treated mice in comparison with those in the control group. TUNEL assay (**d**) was also performed to determine the level of apoptosis. There was significant increase in apoptotic levels in Tan IIA-treated tumor tissues in comparison with the control. Values are mean ± SD (n = 15). An asterisk demonstrates the significant difference (p < 0.05) between control and Tan IIA-treated groups.

The proliferation index determined by cell cycle-related markers, such as antigen KI-67 (Ki-67) and proliferating cell nuclear antigen (PCNA), has prognostic value in cancer patients^23^. Immunohistochemistry (IHC) demonstrated that Tan IIA significantly inhibited Ki67 (Fig. 3a) and PCNA (Fig. 3b) expression in the tumor specimens. During the removal of tumor tissues, we did notice that the bleeding incidence was significantly obvious in the control group, but rare in Tan IIA group. As we know, the decrease in tumor size is correlated with inhibited neovasculization in the tumor. Immunostaining cluster of differentiation 31 (CD31) was used to visualize the formation of microvessel in the tumor mass. The microvessel density in the tumor was markedly reduced in the Tan IIA-treated group compared to the control group (Fig. 3c). The role of apoptosis in the reduction of tumor size was evaluated by terminal deoxynucleotidyl transferase dUTP nick end labeling (TUNEL) assay. The representative results in Fig. 3d clearly demonstrated that more apoptotic cells with deep brown-stained nuclei were observed in the tumors from Tan IIA-treated mice compared to the control group.

### Tan IIA activated the expressions of cysteine-aspartic proteases both *in vivo* and *in vitro*

**Figure 4:**
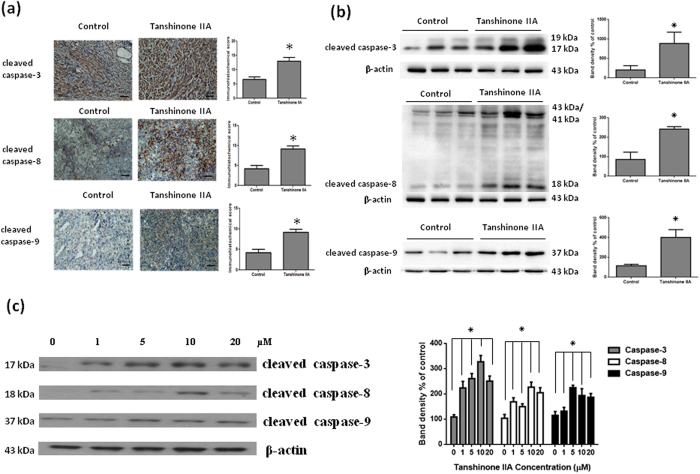
Tan IIA inhibited apoptosis pathway by immunohistochemistry and Western blot both *in vivo* and *in vitro*. The number of positive cells of caspase 3, 8 and 9 (**a**) by IHC and the protein levels of caspase 3, 8 and 9 (**b**) by Western blot from tumor tissue in Tan IIA treatment group was more significantly enhanced than those in control group. 143B cells were treated with the indicated concentrations of Tan IIA for 24 hours. Cell lysates were subjected to Western blot analysis using antibodies including caspase-3, caspase 8, caspase 9 and *β*-actin antibodies. (**c**) All of caspase 3, 8, and 9 were activated by Tan IIA in 143B cells to trigger apoptosis. The bar value is the mean ± SD of three independent experiments in duplicate. An asterisk demonstrates the significant difference (p < 0.05) between control and Tan IIA-treated groups. The Western blot data from cell culture represent one of the three independent experiments.

To determine whether apoptosis plays a critical role during Tan IIA treated osteosarcoma 143B cells both *in vivo* and *in vitro*, we demonstrated apoptosis by investigating downstream caspase 3, 8 and 9 expressions. As shown in Fig. 4a, IHC staining detected increased caspase 3, 8 and 9 expressions in tumors from Tan IIA-treated mice, compared with controls. On the other hand, we further confirmed a significant increase in the protein expression of caspase 3, 8 and 9 in tumors from Tan IIA-treated mice by Western blot in comparison to control group (Fig. 4b). Moreover, the activation of caspase 3, 8, and 9 by Tan IIA in 143B cells were demonstrated (Fig. 4c). These observations indicated that Tan IIA induced cell death both *in vivo* and *in vitro* to limit the growth of tumor mass through an apoptotic pathway.

### Effects of Tan IIA on the expression of Bcl-2 family both *in vivo* and *in vitro*

**Figure 5:**
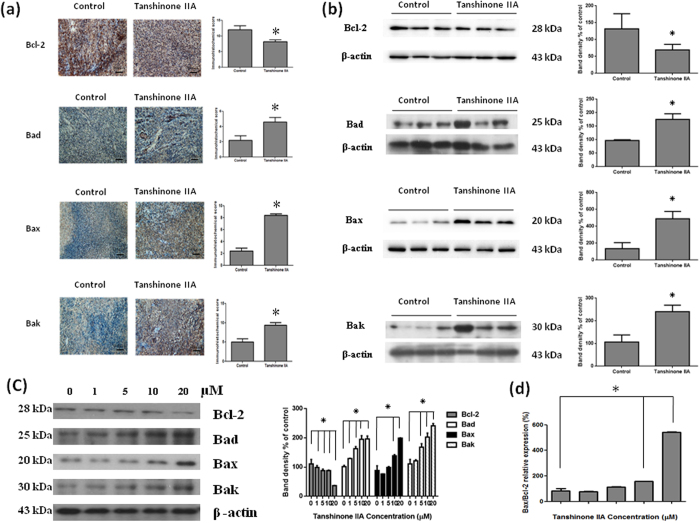
The Bcl-2 family of proteins is critical for the intrinsic mitochondrial apoptotic pathway. They comprise anti-apoptotic (Bcl-2) and pro-apoptotic (including Bad, Bax and Bak) members that function coordinately to control caspase-dependent and -independent cell death. IHC (**a**) and Western blots (**b**) were shown that Bcl-2 was down regulated, on contrary, Bad, Bax and Bak were up regulated significantly in Tan IIA treatment group in comparison with those in none-treated groups. 143B cells were treated with the indicated concentrations of Tan IIA for 24 hours. Cell lysates were subjected to Western blot analysis using antibodies including Bcl-2, Bad, Bax, Bak and β-actin antibodies. (**c**) Tan IIA treatment led to the up regulation of pro-apoptotic protein expression such as Bad, Bax and Bak, while apoptotic protein including Bcl-2 was down regulated in a dose-dependent manner. (**d**) Bax and Bcl-2 ratio was increased. The bar value is the mean ± SD of three independent experiments in duplicate. An asterisk demonstrates the significant difference (p < 0.05) between control and Tan IIA-treated groups. The Western blot data from cell culture represent one of the three independent experiments.

Bcl-2, Bcl-2-associated X protein (Bax), Bcl-2-associated death promoter (Bad) and Bcl-2 homologous antagonist killer (Bak) are Bcl-2 family protein, which is a proto-oncogene best known for its ability to regulate apoptosis. Bax, Bad and Bak are pro-apoptotic molecules. To investigate the regulation of apoptosis by Tan IIA treatment, we analyzed the expression levels of protein with IHC and Western blots both *in vivo* and *in vitro*. We demonstrated a significant decrease in Bcl-2 expression and increase in Bad, Bax and Bak expressions in Tan IIA*-*treated mice were shown by IHC and Western blots (Fig. 5a and b). In addition, Tan IIA treatment of 143B cells led to the up-regulation of pro-apoptotic Bax, Bad and Bak protein expressions, while anti-apoptotic Bcl-2 was down-regulated in a dose-dependent manner (Fig. 5c). The balance of pro-apoptotic Bax and anti-apoptotic Bcl-2 is important in determining whether cells live or die. Bax/Bcl-2 ratio in a cell plays a role in regulating its susceptibility to apoptotic stimuli (Fig. 5d).

### Tan IIA-induced mitochondrial fragmentation related to mitochondrial dynamics and apoptosis in osteosarcoma 143B cells

**Figure 6:**
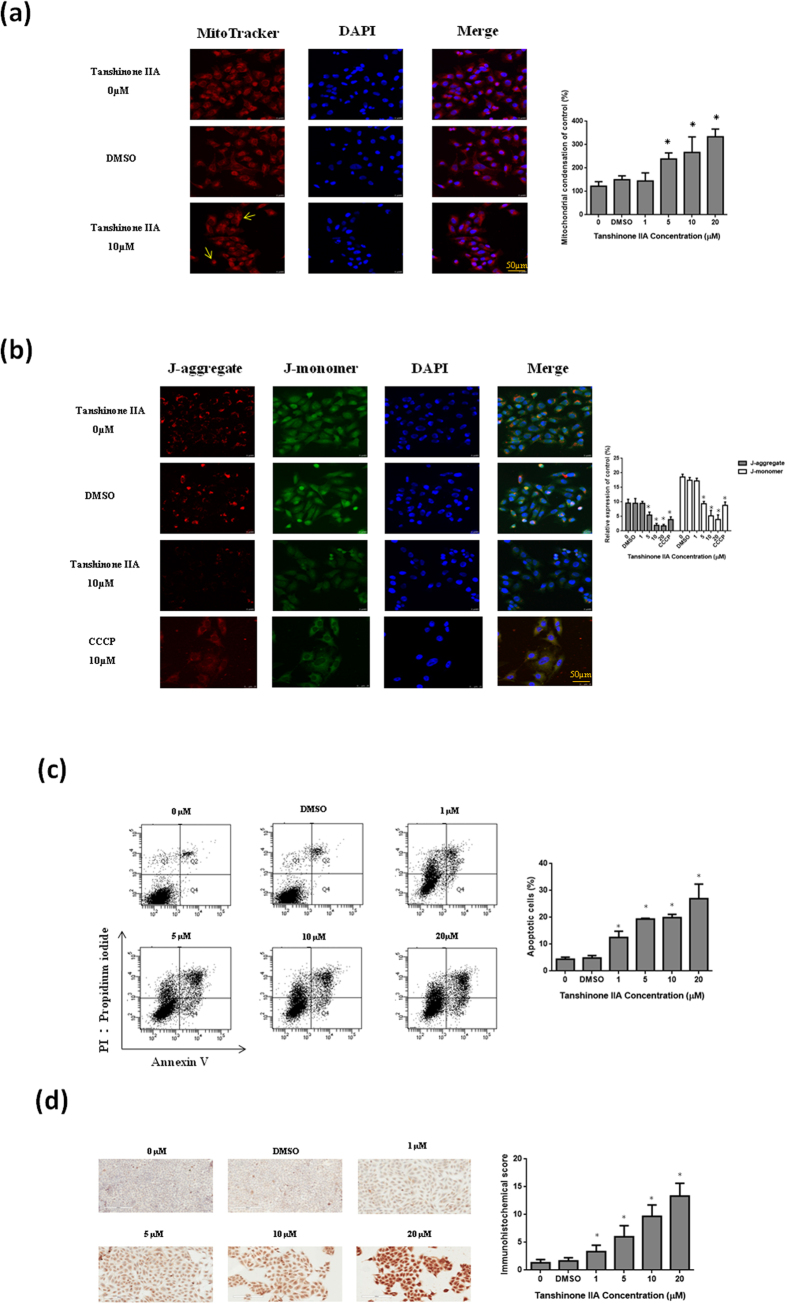
Mitochondrial dynamic changes of morphology and apoptosis in osteosarcoma 143B cells by Tan IIA. Cells treated with 10µM Tan IIA for 24 hours were stained with (**a**) Mito-Tracker red and (**b**) JC-1 to demonstrate mitochondria in live cells. DAPI staining was used to detect all nuclei and visualized under a confocal microscope (200 ×). Tan IIA fragmented the mitochondrial network in the osteosarcoma 143B cells. Annexin V-FITC/propidium iodide staining (**c**) and TUNEL assay (**d**) was also performed to determine the level of apoptosis. There was significant increase in apoptotic levels in 143B cells with the treatment of Tan IIA. The bar value is the mean ± SD of three independent experiments in duplicate. Asterisks mean statistical significance in comparison with the vehicle control (P < 0.05).

**Figure 7:**
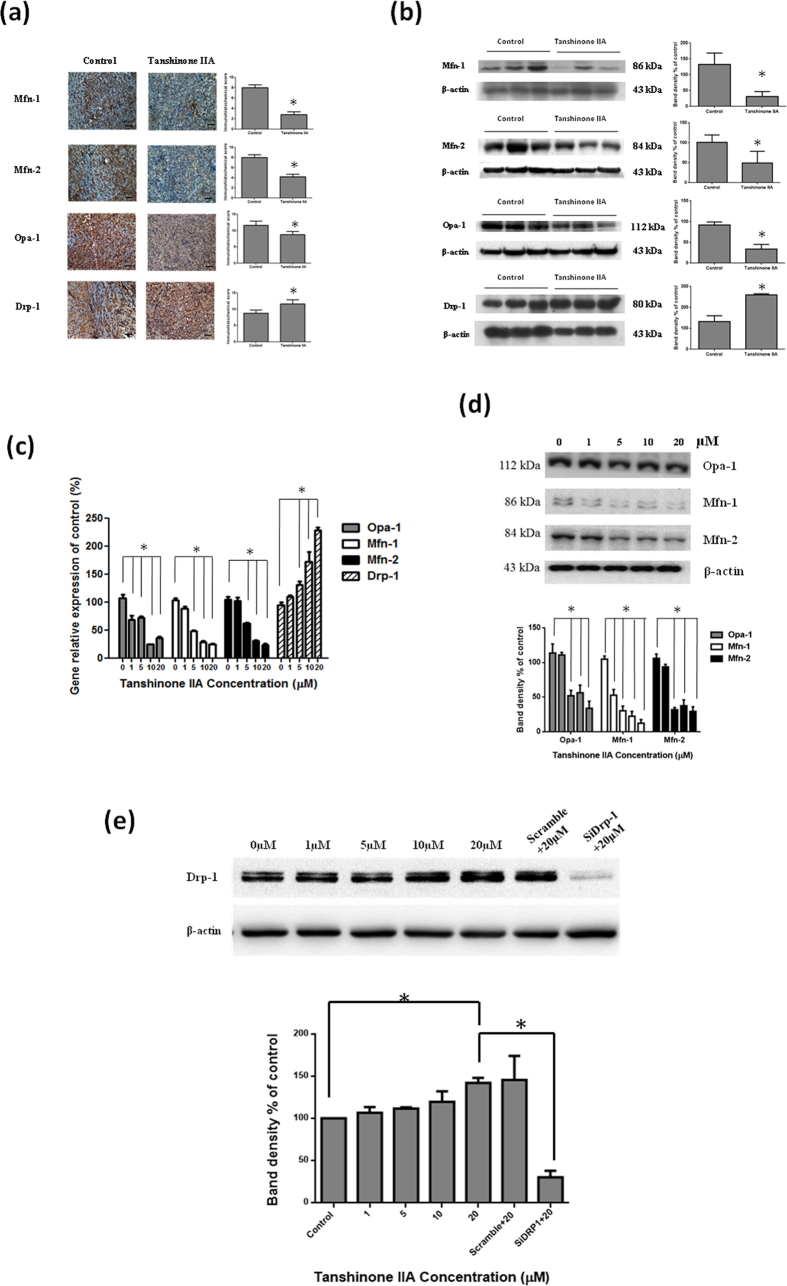
Mitochondrial dynamics includes both fusion and fission mediators to exchange the contents of mitochondria and control mitochondrial morphology. The significant decrease of fusion protein levels including Mfn-1, Mfn-2, and Opa1 in the IHC (**a**) and Western blot (**b**) were noticed in Tan IIA treatment group in comparison with those in none-treated groups. In addition, the significant increase of the fission protein Drp1 was noted in Tan IIA treatment group. 143B cells were treated with the indicated concentrations of Tan IIA for 24 hours. Cell lysates were subjected to Western blot analysis using antibodies such as Mfn-1, Mfn-2, Opa1 and Drp-1 related to mitochondrial dynamics and β-actin as a housekeeping control protein. (**c**) There was a significant decrease in Mfn-1, Mfn-2 and Opa1 related to fusion protein, however, there was a significant increase in Drp-1 related to fission protein. (**d**) Effect of Drp1-siRNA on the cellular Drp1 protein was expressed by immunoblot analysis in 143B cells. They were transfected with Drp1 and control siRNA and co-treated with Tan IIA (20 *μ*M) for 24 h, then harvested for Western blot and normalized with β-actin. Quantification of corresponding densitometric analysis demonstrated Drp1 protein levels in 143B cells. The bar value is the mean ± SD of three independent experiments in duplicate. An asterisk demonstrates the significant difference (p < 0.05) between control and Tan IIA-treated groups. The Western blot data from cell culture represent one of the three independent experiments.

Mitochondria are described as cellular power plants since they create most of the adenosine triphosphate (ATP), which is used as a source of chemical energy. We further investigated dynamic changes of mitochondrial morphology in osteosarcoma 143B cells by Tan IIA treatment. A fluorescent dye, MitoTracker red and JC-1, was used to observe the morphological changes of mitochondria. The mitochondria of osteosarcoma 143B cells without treatment exhibited tubular networks. After incubation with Tan IIA, the mitochondria became shorter and smaller in size, indicating mitochondrial fragmentation (Fig. 6a and b). The mitochondrial membrane potential in 143B osteosarcoma cells was measured by cytofluorimetric analysis with JC-1. The result of 143B cells treated with Tan IIA at various concentrations demonstrated a dose-dependent increase of the blue fluorescence intensity (JC-1-monomers) and a concomitant decrease of the signal with green fluorescence intensity (JC-1-aggregates) as shown in Fig. 1S. It indicated that Tan IIA had the effect to reduce mitochondrial membrane potential to induce apoptosis. Meanwhile, we further examined Tan IIA on apoptotic cell death with annexin V-FITC/propidium iodide staining and TUNEL assay. Our results demonstrated that Tan IIA treatment had the apoptotic effect to induce cell death with dose-dependent manner (Fig. 6c and d). Mitochondrial dynamics includes fusion and fission of mitochondria, exchange the mitochondrial contents as well as morphological changes. IHC and Western blots were used to evaluate the protein levels of these factors in tumors from mice treated with or without Tan IIA. A significant decrease of Mfn-1, Mfn-2, and Opa1 and increase of Drp1 was shown in tissues from Tan IIA-treated mice (Fig. 7a and b). The mRNA levels of mitochondrial fusion-related genes, Mfn-1, Mfn-2 and Opa1 as well as mitochondrial fission-regulating gene, Drp1, were measured. The mRNA of Mfn-1, Mfn-2, and Opa-1 were decreased, while the mRNA levels of Drp-1 were significant increased (Fig. 7c). Fusion proteins, Mfn-1, Mfn-2, and Opa1, were significantly suppressed by Tan IIA treatment, whereas a significant increase of the fission protein, Drp1, was increased (Fig. 7d).To validate the critical role of Drp1 in osteosarcoma 143B cells, RNA interference strategy was employed to investigate the consequence of Drp1 knockdown on the Western blot. Our results demonstrated that transfection of the Drp1-siRNA significantly inhibited endogenous Drp1 protein expression activated by Tan IIA in comparison with the cells treated with Tan IIA in the presence of scrambled siRNA (Fig. 7e). It indicated that Tan IIA modulated mitochondrial dynamics through fusion and fission balance to control morphological changes.

## Discussion

Tan IIA, a diterpenequinonic compound extracted from Danshen, has been shown to inhibit growth and induce apoptosis in various cancer cell lines, including human hepatoma^24^,^25^, breast cancer^16^,^26^, lung cancer^27^,^28^, and leukemia^29^,^30^. However, its inhibitory effect on the most common primary malignant bone tumor, osteosarcoma, is rarely mentioned. Osteosarcoma is the most common malignant bone tumor arising from primitive mesenchymal bone-forming cells with its histologic hallmark, production of malignant osteoid. The main therapy in the patients with osteosarcoma is surgical removal with chemotherapy, for micrometastasis, which is present but often not detectable in most patients (about 80%) at the time of diagnosis^31^.

Recently, Tan IIA has been reported to cause decreased nucleophosmin and prohibitin expression in nuclear matrix and induce nucleophosmin and prohibitin translocation from nucleolus to nucleoplasm and cytoplasm^32^,^33^. These effects were modulated by oncogenes, c-myc and c-fos, as well as tumor suppressor genes, P53 and Rb, in osteosarcoma MG-63 cells. Zhang *et al*.^22^ demonstrated that Tan IIA inhibits the proliferation, migration, and invasion of the osteosarcoma MG-63 cells through the reduction of Bcl-2, MMP-2, and MMP-9 levels; whereas to activates caspase cascades to induce apoptosis. Herein, Tan IIA not only inhibited the proliferation, migration, and invasion of the osteosarcoma 143B cells, but also suppressed the tumor formation and development in NOD-SCID mice implanted with osteosarcoma 143B cells. The average tumor size and weight were significantly reduced in the Tan IIA-treated mice, compared to control. These observations clearly indicate the anti-tumor effect of Tan IIA in osteosarcoma. No obvious toxicity was detected with the Tan IIA treatment, as shown by no significant differences of body weight or histological appearance in major organs including liver, lungs, spleen, kidneys and heart. Although143B cells are known to exhibit metastatic potential, no metastasis was detected in mice treated with or without Tan IIA, possibly due to subcutaneous instead of intravenous injection or intramedullary injection to the tibia. Moreover, proliferation markers, such as Ki67 and PCNA, were significantly decreased in the Tan IIA*-*treated group, compared to controls. Angiogenesis is suggested to be critical for a solid tumor reach few millimeters in size^3^. The inhibition of microvessel formation resulting from limited supply of nutrients and oxygen is therefore responsible for reducing tumor size in Tan IIA-treated mice, as confirmed by CD31 immunostaining.

Apoptosis induced by Tan IIA was further detected by TUNEL assay. The balance between pro-apoptotic (Bax, Bad and Bak) and anti-apoptotic proteins (Bcl-2, Bcl-xL and Mcl-1) controls the sensitivity of cells to apoptotic stimuli^34^. The translocation of apoptotic proteins from the cytosol to the surface of the mitochondria leads to the release of cytochrome c to disrupt transmembrane potential and open the mitochondrial permeability transition pores that is essential to maintain the integrity for production of energy (ATP) and preservation of cellular homeostasis^35^. In the current study, Tan IIA decreased Bcl-2 expression and increased caspase 3 expression, accompanied by an elevated Bax, Bad and Bak expression in osteosarcoma 143B cells and osteosarcoma 143B xenograft mice. The consistent *in vitro* and *in vivo* induction of caspases 3, 8 and 9 with the regulation of Bcl-2 family proteins by Tan IIA suggests that intrinsic pathway is involved the induction of apoptosis in osteosarcoma. Whether the extrinsic death receptor-mediated pathway plays a role in the Tan IIA-induced apoptosis in osteosarcoma cells cannot be determined by this study.

Recently, mitochondrial fusion/fission has been suggested to be associated with the induction of apoptosis through the intrinsic pathway. Bax, Bad and Bak are shown to be downstream to the mitochondrial fission/fusion proteins to initiate caspase activation in the cytosol^10^. Because mitochondrial morphology is tightly controlled by the balance of mitochondrial fission and fusion, we proposed that Tan IIA-induced apoptosis through intrinsic pathway resulted from enhanced fission, reduced fusion, or both. Shenouda *et al*. reported that endothelial expression of Drp1 and Fis1 increased and Opa1 decreased in diabetes mellitus with alterations in mitochondrial networks, ROS production, endothelial nitric oxide synthase (eNOS) activation, and cyclic guanosine monophosphate (cGMP) production^36^. Our findings with decrease in tumor size and IHC analysis of CD31 suggested that Tan IIA might inhibit angiogenesis associated with down regulation of ROS^37^,^38^ through mitochondrial dynamics. In the present study, we further confirmed that Tan IIA indeed reduced the levels of Mfn-1, Mfn-2 and Opa-1 and increased the levels of Drp-1 in both osteosarcoma 143B cells and 143B cell xenograft mice. Together with the up-regulation of Bax, Bad and Bak and down-regulation of Bcl-2, these observations indicated that Tan IIA may affect mitochondrial dynamics through the differential modulation of mitochondrial fission and fusion.

**Figure 8:**
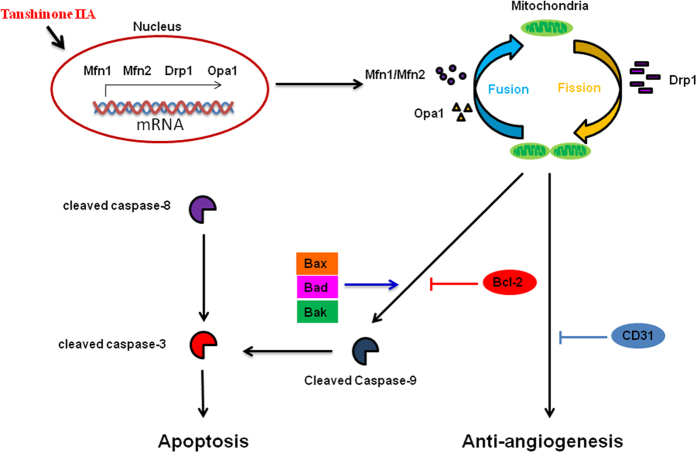
Schematic diagram illustrates the molecular mechanism that Tan IIA induces apoptosis and angiogenesis through mitochondrial dynamics in a human osteosarcoma xenograft mouse model. A variety of physiological death signals, as well as pathological cellular insults, trigger the genetically programmed cell death. Tan IIA induced apoptotic change of osteosarcoma 143B cells through the caspase pathway associated with mitochondrial dysfunction.

In summary, the current study suggests for the first time that Tan IIA induces apoptosis via intrinsic pathways and results in mitochondrial dysfunction and suppresses angiogenesis in osteosarcoma cells using both *in vitro* and *in vivo* models. The possible signaling pathways involved in mitochondrial dynamics As shown in Fig. 8, Tan IIA can act as a cytotoxic agent to induce apoptosis to inhibit tumor growth via the induction of pro-apoptotic proteins and the inhibition of anti-apoptotic molecules. In addition, the balance of mitochondrial fission/fusion and the regulation of tumor angiogenesis are also involved in the novel anti-cancerous effects of Tan IIA. However, further studies are needed to provide a novel strategy to deliver Tan IIA effectively for osteosarcoma therapy.

## Materials and Methods

### Cell Culture

The human osteosarcoma cancer cell line 143B was purchased from the American Type Culture Collection (ATCC) and maintained in Dulbecco’s Modified Eagle’s Medium (DMEM) supplemented with 10% fetal bovine serum (FBS). Cells were maintained in a humidified atmosphere with 5% CO_2_, 95% air at 37 °C. To examine the morphological and cytotoxic effect by Tan IIA, cells at 70% confluence were treated with 0~100 μM of Tan IIA for 24 hrs.

### Animal

All experiments were approved by Institutional Animal Care and Use Committee (Approval No. 2013022001) at Kaohsiung Chang Gung Memorial Hospital (Kaohsiung, Taiwan). The protocols and methods were carried out in accordance with the relevant guidelines as shown in the Guide for the Care and Use of Laboratory Animals as promulgated by the Institute of Laboratory Animal Resources, National Research Council (USA). NOD-SCID (NOD CB17-Prkdcscid/NcrCrl, male, 5 weeks of age) mice were obtained from BioLASCO Taiwan Co., Ltd. All mice were housed under a setting of 12 hours light/dark cycle at 22 ± 1 °C, 55% humidity and fed with water and food provided at regular time. During the entire maintenance period, all mice were permitted free cage activity without joint immobilization. The initial body weights of the mice were between 20 and 23 grams. After subcutaneous injection of 2 × 10^6^ 143B osteosarcoma cells into the back of NOD-SCID mice, the mice were treated with or without Tan IIA (20 mg/kg). Tan IIA was diluted in DMSO: Methanol: Hydroxypropyl-beta-cydodextrin (HP-beta-CD) = 1:1:1. Seven days after 143B osteosarcoma cell injection, IP injection with Tan IIA was carried out every other day followed by sacrifice at day 45 of tumor cell inoculation. A complete autopsy was performed by obtaining the major tissues including tumor mass, liver, kidneys, heart, spleen, and lungs dissected. The weights of major organs as well as total body weight were measured. All experiments were carried out using 5 mice each group in three independent experiments of a time-dependent manner with 3 time points.

### Cell viability by MTT assay

Human osteosarcoma 143B cells with or without Tan IIA treatment were washed once with PBS, followed by adding 1 mL of DMEM containing 0.05 mg/mL of 3-(4,5-dimethylthiazol-2-yl)-2 and 5-diphenyltetrazolium bromide (MTT; Sigma). After incubation at 37 °C for 1 h, the media were removed and the formazan crystals in the cells were dissolved in 1 mL of DMSO for optical density (OD) reading at 570 nm using a spectrophotometer.

### Transwell filter migration assay

Transwell filters (Costar, Cambridge, MA) with 8.0 μm pores were used for migration assay. Osteosarcoma 143B cells cultured without or with treatment of Tan IIA for 24 h were seeded at a density of 1.2 × 10^5^ cells/filter. To initiate the migration assay, cells in 250 μL of M199 without FBS were added to the inner chamber and the lower chamber was filled with 600 μl of M199 with 10% FBS as an inducer of cell migration. Cells were allowed to migrate for 2 h at 37 °C in an atmosphere of 95% air/5% CO_2_. Cells on the lower surface of the filter were first stained with 1% crystal violet in 2% ethanol for 10 min and cells remaining on the upper surface of the filter were removed using a cotton swab. The cells that migrated onto the lower surface of the filter were examined by microscope after mounting on a slide. A total of six random high-power microscopic fields (HPF) (100X) per filter were photographed.

### *In vitro* invasion assay

Cell invasion assays were performed using Transwells (8-μm pore size, Corning Costar Corp.). A 50-μg aliquot of matrigel solution was placed on the filter surface and incubated at 37 °C for 2 hours to produce an artificial basement membrane. After rinse with phosphate-buffered saline (PBS), the filters were placed into wells of 24-well plate, and 600 μL of DMEM containing10% FBS was added to the lower compartment. Osteosarcoma 143B cells were pretreated with 0, 1, 5, 10 and 20 μM of Tan IIA for 24 h and then added to the upper chamber with100 μL of serum-free DMEM containing 2 × 10^5^ cells. The invasion was carried out for 24 h and osteosarcoma 143B cells on the filter were first stained with 1% crystal violet in 2% ethanol for additional 10 min and cells remaining on the upper surface of the filter were removed by a cotton swab. The cells that invaded onto the lower surface of the filter were examined by microscope after mounting on a slide. A total of six random HPF (100X) per filter were photographed.

### Hematoxylin and eosin stain

For hematoxylin and eosin staining, paraffin-embedded sample slides were de-paraffinized, hydrated, and then stained with hematoxylin for 1 min. After rinse, the slides were stained with eosin for 5 min, rinsed, and sealed with cover slips. Tissue sections from tumor mass, kidneys, spleen, liver, heart and lungs were used. The slides were counterstained with hematoxylin and mounted. All slides were examined under light microscopy.

### Immunohistochemistry (IHC)

The slides were de-waxed in xylene, hydrated using graded ethanol and washed in PBS. The tissue sections were incubated in 3% H_2_O_2_/methanol for 30 min and then briefly heated in citrate buffer, using autoclave, for 15 min. The blocking step was performed before application of the primary antibody with 5% bovine serum albumin. After blocking, the sections were incubated overnight at 4 °C with anti-mouse Ki-67, Bak, Bcl2 (Millipore, Bedford, MA, USA), PCNA, CD31, cleaved Caspase-8 (Cell Signaling, Danvers, MA, USA), cleaved Caspase-3 (GeneTex, Irvine, CA, USA), cleaved Caspase-9 (Origene, Rockville, MD, USA), Bad (SignalChem, Tirana, Albania), Bax, Mfn1, Mfn2, Opa1, and DRP1 (abcam, Cambridge, UK) antibodies. The sections were then exposed to the corresponding secondary antibodies. The antigen-antibody complex was detected by 3,3′-diaminobenzidine (DAB) with Mayer’s hematoxylin counterstaining. All slides were examined under light microscopy. We calculated IHC scores according to positive cells and intensity of staining product as described in previous report^39^.

### Annexin V-FITC/propidium iodide staining

143B cells in the presence or absence of Tan IIA were harvested and washed twice with cold PBS, then resuspended in 1× binding buffer at a concentration of 6 × 105 cells/mL. 100 μL of the solution (6 × 104 cells) was then transferred to a 5 mL culture tube. All samples were processed for annexin V labeling according to the manufacturer’s instructions. Briefly, cells were resuspended in 100 μL of 1× annexin V binding buffer (BD Biosciences) and were fluorescently labeled for the simultaneous detection of apoptotic and necrotic cells by adding 5 μL of annexin V-FITC (BD Biosciences) and 5 μL of 1 mg/mL propidiumiodide (PI) to each sample. Samples were gently mixed and incubated at room temperature in the dark for 30 min. At the end of the incubation, 400 μg of 1× binding buffer was added to each sample and the sample was analyzed using a flow cytometer (Becton Dickinson) equipped with CellQuest software.

### TUNEL assay

*In-Situ* Cell Death Detection Kit (ROCHE, Indianapolis, IN USA)for TUNEL was used. The slides were dewaxed in xylene, hydrated by graded ethanol. De-paraffinized sections were washed with PBS and incubated with proteinase K for 30 min at 37 °C. After washing three times with PBS, sections were treated with a terminal deoxynucleotidyltransferase (TdT), incubated with 3% hydrogen peroxide for 5 min to block endogenous peroxidase activity, and then treated with peroxidase conjugated antibody for 10 min at room temperature. After washing in PBS, nick end labeling was visualized by immersing sections in DAB solution with 3% hydrogen peroxide and counterstained with methyl green. Finally, the sections were counterstained with Mayer’s hematoxylin and washing in water and mounted. All slides were examined under light microscopy.

### Western blot analysis

The tumor masses and 143B cells cultured with or without Tan IIA were harvested and total cell protein was extracted using whole cell lysis buffer. The protein concentrations were determined by the Bradford method (Bio-Rad, CA, USA). Samples with equal amount of protein were subjected to 8–15% sodium dodecyl sulfate polyacrylamide gel electrophoresis (SDS-PAGE) and transferred onto a polyvinylidenedifluoride (PVDF) (Millipore, Bedford, MA, USA) membrane. The membrane was incubated at room temperature in blocking solution (10% nonfat milk) for 1 h followed by incubation for 2 h in blocking solution containing an appropriate dilution (1:1000) of rabbit anti-cleaved caspase-3, -cleaved caspase-8, -cleaved caspase-9, -Bax, -Bcl-2, Bad, -Bak, -Mnf1, -Mnf2, -Opa1, or -Drp1 antibody (Cell Signaling Technology, Danvers, MA, USA). After washing, the membrane was incubated in PBS containing goat anti-rabbit IgG conjugated with horseradish peroxidase (Sigma, St. Louis, MO, USA) for 1 h. The membrane was washed and autoradiography of the membranes was processed using an enhanced chemiluminescence (ECL) system (Amersham Pharmacia Biotech, Pittsburgh, PA, USA). Membranes were exposed to Fuji medical X-ray film (Fuji Ltd., Tokyo, Japan) for 30 min. The β-actin expression was used as the internal control.

### Mitochondrial staining and transfection plasmid with immunofluorescence

Osteosarcoma 143B cells were seeding in triplicate at a density of 7 × 10^5^ cells/well in 12-well plates. Cells were transfected with PCDNA 3.1(+)-mito-Ds Red plasmid (offered by Prof. David Chen from Caltech, CA, USA) in 50 μL of Opti-MEMmedium. Then, 5 μL of RNAi MAX (Invitrogen, Waltham, MA, USA) was gently mixed with 50 μL of Opti-MEM medium and incubated for 5 min at room temperature. After incubation, the diluted DNA with the diluted RNAiMAX (total volume 100 μL) was combined and incubated for another 20 min at room temperature to allow the DNA- RNAi MAX (Invitrogen, Waltham, MA, USA) complexes to form. The 100 μL of DNA-RNAi MAX complexes of each well were mixed gently by rocking the plate back and forth and incubated subsequently, the plates were gently mixed and incubated at 37 °C in a CO_2_ incubator for 4–6 h. Cells plated on slides were treated with or without various concentrations of Tan IIA (1, 5, 10, 20 μM) for 24 hours and stained with 5 μM MitoTracker red (Invitrogen, Waltham, MA, USA) or 10 μg/mL JC-1 (Thermo Fisher Scientific, Waltham, MA, USA) in DMEM medium at 37 °C for 30 min. Cells were stained with DAPI. After mounting in fluoromount media (Sigma-Aldrich Co. LLC, St. Louis, MO, USA), the slides were visualized under a confocal microscopy (200X) and the images were recorded.

### RNA isolation and quantitative reverse transcription-polymerase chain reaction (qRT-PCR)

Total RNA was extracted by a single step phenol–chloroform–isoamyl alcohol extraction procedure modified from the protocol previously described^3^. Briefly, untreated or cells treated with Tan IIA were harvested and lysed in 4 M guanidinium isothiocyanate buffer containing 25 mM sodium citrate (pH 7.0), 0.5% sodium sarkosine and 0.1 M β-mercaptoethanol. Subsequently, 1/10 volume of 2 M sodium acetate (pH 4.0), one volume of phenol and 1/5 volume of chloroform-isoamyl alcohol (49:1, v:v) were added to the homogenate. After vigorous vortexing for 30 seconds, the solution was centrifuged at 10,000 × g for 15 min at 4 °C. After removing aqueous phase, RNA was precipitated by the addition of 0.5 mL isopropanol. For real-time PCR analysis, reverse transcription was performed using 1 μg of total RNA and oligo (dT) primer in a 20 μl reaction according to the manufacture’s protocol (Applied Biosystems, Foster City, California, USA). Real-time PCR was performed using the Mx3005 qPCR system (Stratagene, La Jolla, CA, USA) with SYBR green (Applied Biosystems, Foster City, California, USA). The PCR was carried out by initial denaturation at 95 °Cfor 2 min and 40 cycles of after): 1) denaturation, 95 °C, 15 seconds; 2) annealing and extension, 60 °C, 1 min. Sequences for the specific primers used in the PCR are Drp-1 Forword (5′-CTGACGCTTGTGGATTTACC-3′) and Reverse (5′-CCCTTCCCATCAATACATCC-3′); Mfn-1Forword (5′-GATTGGCGTCCGTTACATCT-3′) and Reverse (5′-AGTTTCCAGCCTATAGTTTTCCAA-3′); Mfn-2 Forword (5′-TCAGCTACACTGGCTCCAAC-3′) and Reverse (5′-CAAAGGTCCCAGACAGTTCC-3′); Opa-1 Forword (5′-GCAGGATTCAGCAGATAA-3′) and Reverse (5′-CTCTTCTTCATATTCTCTTATAGC-3′); GAPDH Forword (5′-TTCATTGACCTCAACTACAT-3′) and Reverse (5′-GAGGGGCCATCCACAGTCTT-3′).

### Liposome-mediated transfection of 143B cells with siRNA

143B cells were seeding in triplicate at a density of 7 × 10^5^ cells per well in 12-well plates. After overnight incubation, cells were transfected with siRNA Non-targeting siRNA and Drp1 siRNA (20 μM) in 50 μL of Opti-MEM medium. Then, 5 μL of RNAi MAX (Invitrogen) was gently mixed with 50 μL of Opti-MEM medium and incubated for 5 min at room temperature. After the 5 min incubation, the diluted DNA with the diluted RNAi MAX (total volume 100 μL) was combined and incubated for another 20 min at room temperature to allow the DNA-RNAi MAX (Invitrogen) complexes forming. The 100 μL of DNA-RNAi MAX complexes of each well were mixed gently by rocking the plate back and forth and incubated at 37 °C in a CO_2_ incubator for 4–6 hours. After overnight incubation, cells were treated with or without Tan IIA for 24 hours. The cells were washed two times with PBS and collected protein, continued according to western blot analysis methods. The β-actin expression was used as the internal control.

### Statistical analysis

All statistical analyses were performed using SigmaStat statistical software (version 2.0, Jandel Scientific, CA, USA). Results were represented as means ± standard deviation (SD). One-way ANOVA was carried out when multiple comparisons were evaluated. Values were considered to be significant at a P value less than 0.05. All experiments were repeated at least three times independently.

## Additional Information

**How to cite this article:** Huang, S.-T. *et al*. Tanshinone IIA induces intrinsic apoptosis in osteosarcoma cells both *in vivo* and *in vitro* associated with mitochondrial dysfunction. *Sci. Rep.* **7**, 40382; doi: 10.1038/srep40382 (2017).

**Publisher's note:** Springer Nature remains neutral with regard to jurisdictional claims in published maps and institutional affiliations.

## Supplementary Material

Supplementary Information
