# Supplementary material for: Tanshinone IIA induces intrinsic apoptosis in osteosarcoma cells both in vivo and in vitro associated with mitochondrial dysfunction
Source: Sci Rep. 2017 Jan 20;7:40382. doi: 10.1038/srep40382 (PMC5247764; doi:10.1038/srep40382)
Supplement: Supplementary Information [file srep40382-s1.doc]

**Tanshinone IIA induces intrinsic apoptosis in osteosarcoma cells both in vivo and in vitro associated with mitochondrial dysfunction**

Sheng-Teng Huang^1,2,*^, Chao-Chun Huang^3^, Wen-Liang Huang^1,2^, Tsu-Kung Lin^4^, Pei-Lin Liao^5^, Pei-WenWang^6^, Chia-Wei Liou^4^, Jiin-Haur Chuang^5^

**Supplementary Figure S1**: Schematic diagram represented the presence of JC-1 aggregates (red fluorescence; viable cells) and JC-1 monomers (green fluorescence; dead cells) as a result of 143B cells treated with Tan IIA at various concentrations for 24 h, stained with JC-1 and followed by quantitative analysis by flow cytometry. The left side indicating normal mitochondria was decreased with dose dependent manner, whereas the right side indicating depolarized mitochondria was increased dose dependently. CCCP, carbonyl cyanide 3-chlorophenylhydrazone (positive control)
